# (*E*)-Ethyl *N*′-(3-hy­droxy­benzyl­idene)hydrazinecarboxyl­ate dihydrate

**DOI:** 10.1107/S1600536811025104

**Published:** 2011-07-02

**Authors:** Xian-Chao Hu, Jie Zhang, Da-Yong Yang, Lu-Ping Lv

**Affiliations:** a College of Chemical Engineering and Materials Science, Zhejiang University of Technology, Hangzhou 310014, People’s Republic of China; b Research Center of Analysis and Measurement, Zhejiang University of Technology, Hangzhou 310014, People’s Republic of China; c Hangzhou Fist Chemical Co. Ltd, Xiaoshan, Hangzhou 310007, People’s Republic of China; d Linjiang College, Hangzhou Vocational and Technical College, Hangzhou 310018, People’s Republic of China

## Abstract

The asymmetric unit of the title compound, C_10_H_12_N_2_O_3_·2H_2_O, contains two organic mol­ecules with similar conformations and four water mol­ecules. Each organic mol­ecule is close to planar (r.m.s. deviations = 0.035 and 0.108 Å) and adopts a *trans* conformation with respect to its C=N bond. In the crystal, the components are linked into a three-dimensional network by N—H⋯O, O—H⋯O, O—H⋯N and C—H⋯O hydrogen bonds, some of which are bifurcated. An *R*_2_^2^(8) loop occurs between adjacent organic mol­ecules.

## Related literature

For general background to benzaldehyde­hydrazone derivatives, see: Parashar *et al.* (1988 ▶); Hadjoudis *et al.* (1987 ▶); Borg *et al.* (1999 ▶). For a related structure, see: Shang *et al.* (2007 ▶).
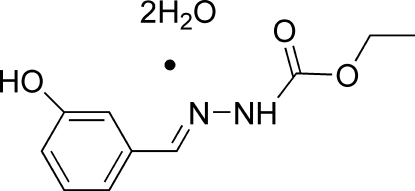


## Experimental

### 

#### Crystal data

C_10_H_12_N_2_O_3_·2H_2_O
*M* *_r_* = 244.25
Monoclinic, 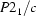

*a* = 12.8074 (10) Å
*b* = 21.9101 (18) Å
*c* = 8.9048 (7) Å
β = 96.490 (3)°
*V* = 2482.8 (3) Å^3^
*Z* = 8
Mo *K*α radiation
μ = 0.11 mm^−1^
*T* = 223 K
0.20 × 0.19 × 0.18 mm

#### Data collection

Bruker SMART CCD diffractometer
Absorption correction: multi-scan (*SADABS*; Bruker, 2002 ▶) *T*_min_ = 0.977, *T*_max_ = 0.989
22432 measured reflections
4819 independent reflections
3377 reflections with *I* > 2σ(*I*)
*R*_int_ = 0.036

#### Refinement

*R*[*F* ^2^ > 2σ(*F* ^2^)] = 0.043
*wR*(*F* ^2^) = 0.126
*S* = 0.95
4819 reflections
341 parameters
H atoms treated by a mixture of independent and constrained refinement
Δρ_max_ = 0.16 e Å^−3^
Δρ_min_ = −0.14 e Å^−3^

### 

Data collection: *SMART* (Bruker, 2002 ▶); cell refinement: *SAINT* (Bruker, 2002 ▶); data reduction: *SAINT*; program(s) used to solve structure: *SHELXS97* (Sheldrick, 2008 ▶); program(s) used to refine structure: *SHELXL97* (Sheldrick, 2008 ▶); molecular graphics: *SHELXTL* (Sheldrick, 2008 ▶); software used to prepare material for publication: *SHELXTL*.

## Supplementary Material

Crystal structure: contains datablock(s) I, global. DOI: 10.1107/S1600536811025104/hb5929sup1.cif

Structure factors: contains datablock(s) I. DOI: 10.1107/S1600536811025104/hb5929Isup2.hkl

Supplementary material file. DOI: 10.1107/S1600536811025104/hb5929Isup3.cml

Additional supplementary materials:  crystallographic information; 3D view; checkCIF report

## Figures and Tables

**Table 1:** Hydrogen-bond geometry (Å, °)

*D*—H⋯*A*	*D*—H	H⋯*A*	*D*⋯*A*	*D*—H⋯*A*
N2—H2⋯O6^i^	0.86	2.29	3.0968 (18)	157
N4—H4*N*⋯O3	0.86	2.24	3.0849 (18)	167
O6—H6⋯O4*W*	0.82	1.90	2.675 (2)	157
C3—H3⋯O5	0.93	2.51	3.422 (2)	168
O1*W*—H1*A*⋯O2*W*^ii^	0.84 (3)	2.04 (3)	2.874 (3)	178 (3)
O1*W*—H1*B*⋯O2*W*^iii^	0.91 (3)	1.99 (3)	2.906 (2)	177 (3)
O2*W*—H2*A*⋯O4	0.91 (3)	2.02 (3)	2.899 (2)	163 (2)
O2*W*—H2*B*⋯O1^iv^	0.85 (3)	2.25 (3)	2.9268 (19)	136 (2)
O2*W*—H2*B*⋯N1^iv^	0.85 (3)	2.41 (3)	3.165 (2)	148 (2)
O3*W*—H3*A*⋯O4^iii^	0.89 (3)	2.27 (3)	2.9417 (19)	132 (2)
O3*W*—H3*A*⋯N3^iii^	0.89 (3)	2.38 (3)	3.200 (2)	153 (3)
O3*W*—H3*B*⋯O1	0.90 (3)	2.10 (3)	2.991 (2)	171 (3)
O4*W*—H4*B*⋯O3*W*^v^	0.98 (6)	1.84 (6)	2.819 (3)	173 (5)
O4*W*—H4*A*⋯O3*W*^iv^	0.81 (3)	2.15 (3)	2.955 (2)	169 (3)

Symmetry codes: (i) 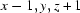; (ii) 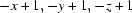; (iii) 
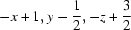
; (iv) 
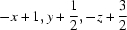
; (v) 
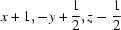
.

**Fig. 1.**
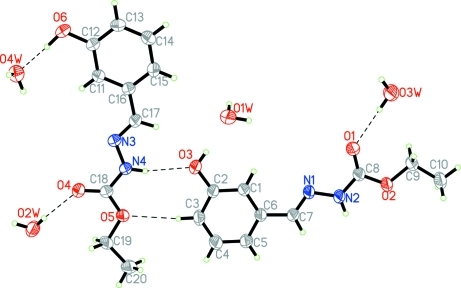
Molecular structure of (I), showing 40% probability displacement ellipsoids and the atomic numbering.

**Fig. 2.**
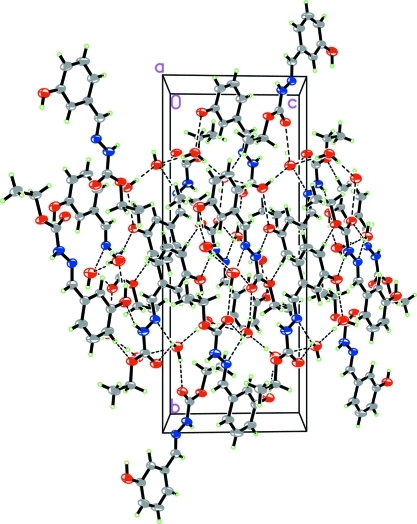
Crystal packing of the title compound, viewed approximately down the a axis. Dashed lines indicate hydrogen bonds. H atoms not intervening in H-bonding were eliminated for clarity.

## supplementary crystallographic information

### Comment

Benzaldehydehydrazone derivatives have received considerable attentions for a long time due to their pharmacological activity (Parashar *et al.*, 1988) and their photochromic properties(Hadjoudis *et al.*, 1987). Meanwhile, it's an important intermidiate of 1,3,4-oxadiazoles, which have been reported to be versatile compounds with many properties (Borg *et al.*, 1999). As a further investigation of this type of derivatives, we report herein the crystal structure of the title compound.

The title compound, C_10_H_12_N_2_O_3_ .2H_2_O, crystallizes with two very similar independent molecules in the asymmetric unit. Each independent molecule adopts a *trans* configuration with respect to the C═N bond. The N1/N2/O1/O2/C7-C10 and N3/N4/O4/O5/C17-C20 planes form dihedral angles of 2.56 (10)° and 8.02 (8)°, respectively, with the C1—C6 and C1—C16 planes. The bond lengths and angles of the main molecule agree with those observed for (E)-Methyl N'-(4-hydroxybenzylidene)hydrazinecarboxylate (Shang *et al.*, 2007).

In the crystal, molecules are linked into three-dimensional network by N—H···O,O—H···O,C—H···O and O—H···N hydrogen bonds (Table 1, Fig.2).

### Experimental

3-hydroxybenzaldehyde (1.22g, 0.01mol) and ethyl hydrazinecarboxylate(1.04g, 0.01mol) were dissolved in stirred methanol (30ml) and left for 3h at room temperature. The resulting solid was filtered off and recrystallized from ethanol to give the title compound in 88% yield.  Colourless blocks of (I) were obtained by slow evaporation of a ethanol solution at room temperature (m.p. 438-441 K).

### Refinement

H atoms of the water molecule were located in a difference map and were refined with O-H distances restrained to 0.81 (3) Å,0.84 (3) Å, 0.85 (3) Å, 0.90 (3) Å, 0.91 (3) Å, 0.95 (3) Åand 0.98 (3) Å, H atoms were included in the riding model approximation with N-H = 0.86Å and O-H=0.82Å. C-bound H atoms were positioned geometrically (C-H = 0.93Å and 0.96Å) and refined using a riding model, with U_iso_(H) = 1.2-1.5U_eq_(C).

### Crystal data

**Table:**

C_10_H_12_N_2_O_3_·2H_2_O	*F*(000) = 1040
*M**_r_* = 244.25	*D*_x_ = 1.307 Mg m^−^^3^
Monoclinic, *P*2_1_/*c*	Mo *K*α radiation, λ = 0.71073 Å
Hall symbol: -P 2ybc	Cell parameters from 4819 reflections
*a* = 12.8074 (10) Å	θ = 1.6–25.0°
*b* = 21.9101 (18) Å	µ = 0.11 mm^−^^1^
*c* = 8.9048 (7) Å	*T* = 223 K
β = 96.490 (3)°	Block, colourless
*V* = 2482.8 (3) Å^3^	0.20 × 0.19 × 0.18 mm
*Z* = 8	

### Data collection

**Table:**

Bruker SMART CCD diffractometer	4819 independent reflections
Radiation source: fine-focus sealed tube	3377 reflections with *I* > 2σ(*I*)
graphite	*R*_int_ = 0.036
φ and ω scans	θ_max_ = 26.0°, θ_min_ = 1.6°
Absorption correction: multi-scan (*SADABS*; Bruker, 2002)	*h* = −15→15
*T*_min_ = 0.977, *T*_max_ = 0.989	*k* = −26→27
22432 measured reflections	*l* = −10→10

### Refinement

**Table:**

Refinement on *F*^2^	Secondary atom site location: difference Fourier map
Least-squares matrix: full	Hydrogen site location: inferred from neighbouring sites
*R*[*F*^2^ > 2σ(*F*^2^)] = 0.043	H atoms treated by a mixture of independent and constrained refinement
*wR*(*F*^2^) = 0.126	*w* = 1/[σ^2^(*F*_o_^2^) + (0.0615*P*)^2^ + 0.604*P*] where *P* = (*F*_o_^2^ + 2*F*_c_^2^)/3
*S* = 0.95	(Δ/σ)_max_ < 0.001
4819 reflections	Δρ_max_ = 0.16 e Å^−^^3^
341 parameters	Δρ_min_ = −0.14 e Å^−^^3^
0 restraints	Extinction correction: *SHELXL97* (Sheldrick, 2008), Fc^*^=kFc[1+0.001xFc^2^λ^3^/sin(2θ)]^-1/4^
Primary atom site location: structure-invariant direct methods	Extinction coefficient: 0.0022 (6)

### Special details

**Table:**

Geometry. All esds (except the esd in the dihedral angle between two l.s. planes) are estimated using the full covariance matrix. The cell esds are taken into account individually in the estimation of esds in distances, angles and torsion angles; correlations between esds in cell parameters are only used when they are defined by crystal symmetry. An approximate (isotropic) treatment of cell esds is used for estimating esds involving l.s. planes.
Refinement. Refinement of F^2^ against ALL reflections. The weighted R-factor wR and goodness of fit S are based on F^2^, conventional R-factors R are based on F, with F set to zero for negative F^2^. The threshold expression of F^2^ > 2sigma(F^2^) is used only for calculating R-factors(gt) etc. and is not relevant to the choice of reflections for refinement. R-factors based on F^2^ are statistically about twice as large as those based on F, and R- factors based on ALL data will be even larger.

### Fractional atomic coordinates and isotropic or equivalent isotropic displacement parameters (Å^2^)

**Table:**

	*x*	*y*	*z*	*U*_iso_*/*U*_eq_	
C10	−0.09028 (17)	0.12370 (9)	1.3239 (3)	0.0742 (6)	
H10A	−0.1020	0.0806	1.3319	0.111*	
H10B	−0.1545	0.1434	1.2842	0.111*	
H10C	−0.0670	0.1400	1.4221	0.111*	
O1W	0.42004 (14)	0.30255 (7)	0.7045 (2)	0.0734 (5)	
O2W	0.68695 (13)	0.73071 (8)	0.58510 (19)	0.0723 (4)	
O3W	0.14922 (13)	0.05421 (8)	1.0133 (2)	0.0770 (5)	
O4W	0.98022 (17)	0.48067 (8)	0.3000 (2)	0.0847 (5)	
H2A	0.694 (2)	0.6903 (13)	0.607 (3)	0.100 (9)*	
H1A	0.387 (2)	0.2930 (13)	0.621 (4)	0.112 (12)*	
H1B	0.386 (2)	0.2811 (13)	0.772 (3)	0.114 (10)*	
H2B	0.745 (2)	0.7398 (11)	0.554 (3)	0.096 (9)*	
H3A	0.213 (3)	0.0450 (13)	0.989 (3)	0.125 (11)*	
H4A	0.938 (3)	0.5007 (15)	0.341 (3)	0.122 (12)*	
H3B	0.150 (2)	0.0953 (15)	1.023 (3)	0.133 (12)*	
H4B	1.041 (5)	0.472 (3)	0.374 (6)	0.28 (3)*	
C1	0.28335 (13)	0.39669 (8)	0.9118 (2)	0.0477 (4)	
H1	0.2871	0.3558	0.8849	0.057*	
C2	0.34613 (13)	0.43925 (8)	0.8508 (2)	0.0507 (4)	
C3	0.33908 (16)	0.49999 (8)	0.8888 (3)	0.0667 (6)	
H3	0.3813	0.5287	0.8480	0.080*	
C4	0.26972 (18)	0.51811 (9)	0.9868 (3)	0.0793 (7)	
H4	0.2646	0.5592	1.0112	0.095*	
C5	0.20760 (16)	0.47581 (8)	1.0491 (3)	0.0670 (6)	
H5	0.1608	0.4884	1.1155	0.080*	
C6	0.21479 (13)	0.41483 (7)	1.0130 (2)	0.0480 (4)	
C7	0.14945 (14)	0.37163 (8)	1.0848 (2)	0.0536 (5)	
H7	0.1046	0.3868	1.1511	0.064*	
C8	0.07698 (13)	0.22053 (8)	1.1273 (2)	0.0506 (4)	
C9	−0.00809 (14)	0.13484 (8)	1.2203 (2)	0.0547 (5)	
H9A	0.0575	0.1155	1.2595	0.066*	
H9B	−0.0303	0.1183	1.1207	0.066*	
C11	0.77878 (13)	0.39363 (8)	0.4056 (2)	0.0483 (4)	
H11	0.7901	0.4350	0.3906	0.058*	
C12	0.83693 (13)	0.35064 (8)	0.3377 (2)	0.0513 (4)	
C13	0.81986 (16)	0.28929 (9)	0.3588 (3)	0.0669 (6)	
H13	0.8588	0.2605	0.3125	0.080*	
C14	0.74537 (18)	0.27088 (9)	0.4480 (3)	0.0791 (7)	
H14	0.7341	0.2295	0.4623	0.095*	
C15	0.68699 (16)	0.31331 (8)	0.5169 (3)	0.0669 (6)	
H15	0.6365	0.3004	0.5773	0.080*	
C16	0.70352 (13)	0.37501 (8)	0.4961 (2)	0.0483 (4)	
C17	0.64060 (14)	0.41813 (8)	0.5720 (2)	0.0529 (5)	
H17	0.5858	0.4029	0.6212	0.063*	
C18	0.59999 (13)	0.56870 (8)	0.6705 (2)	0.0478 (4)	
C19	0.52938 (14)	0.65317 (8)	0.7918 (2)	0.0552 (5)	
H19A	0.5111	0.6780	0.7025	0.066*	
H19B	0.5992	0.6646	0.8366	0.066*	
C20	0.45141 (16)	0.66243 (9)	0.9025 (3)	0.0696 (6)	
H20A	0.4511	0.7046	0.9317	0.104*	
H20B	0.4704	0.6376	0.9902	0.104*	
H20C	0.3827	0.6510	0.8567	0.104*	
N1	0.15055 (11)	0.31441 (6)	1.06170 (17)	0.0508 (4)	
N2	0.08175 (12)	0.28158 (7)	1.13816 (19)	0.0604 (4)	
H2	0.0413	0.3004	1.1936	0.073*	
N3	0.65632 (10)	0.47535 (6)	0.57456 (16)	0.0480 (4)	
N4	0.58747 (11)	0.50831 (6)	0.65046 (18)	0.0543 (4)	
H4N	0.5359	0.4901	0.6854	0.065*	
O1	0.12881 (11)	0.18908 (6)	1.05295 (17)	0.0687 (4)	
O2	0.00520 (9)	0.20017 (5)	1.21202 (15)	0.0578 (4)	
O3	0.41697 (10)	0.42375 (6)	0.75455 (16)	0.0694 (4)	
H3C	0.4154	0.3867	0.7409	0.104*	
O4	0.66657 (10)	0.59992 (5)	0.62166 (16)	0.0600 (4)	
O5	0.52645 (9)	0.58923 (5)	0.75261 (16)	0.0579 (4)	
O6	0.91245 (10)	0.36631 (6)	0.24827 (17)	0.0721 (4)	
H6	0.9188	0.4035	0.2474	0.086 (8)*	

### Atomic displacement parameters (Å^2^)

**Table:**

	*U*^11^	*U*^22^	*U*^33^	*U*^12^	*U*^13^	*U*^23^
C10	0.0782 (14)	0.0505 (12)	0.1003 (17)	−0.0111 (10)	0.0373 (13)	0.0089 (11)
O1W	0.0912 (11)	0.0552 (9)	0.0780 (12)	0.0013 (8)	0.0280 (11)	−0.0056 (8)
O2W	0.0765 (10)	0.0610 (10)	0.0868 (11)	−0.0007 (8)	0.0417 (9)	0.0052 (8)
O3W	0.0635 (9)	0.0624 (10)	0.1118 (13)	−0.0038 (7)	0.0396 (9)	−0.0021 (9)
O4W	0.0879 (12)	0.0599 (10)	0.1142 (15)	−0.0092 (8)	0.0454 (12)	−0.0058 (9)
C1	0.0481 (9)	0.0356 (9)	0.0620 (11)	−0.0014 (7)	0.0182 (8)	−0.0010 (8)
C2	0.0505 (10)	0.0451 (10)	0.0601 (11)	−0.0003 (8)	0.0220 (8)	0.0027 (8)
C3	0.0721 (13)	0.0414 (11)	0.0938 (16)	−0.0056 (9)	0.0409 (12)	0.0067 (10)
C4	0.0957 (16)	0.0370 (10)	0.1155 (19)	−0.0006 (10)	0.0575 (15)	−0.0041 (11)
C5	0.0732 (13)	0.0435 (11)	0.0930 (16)	0.0030 (9)	0.0476 (12)	−0.0037 (10)
C6	0.0463 (9)	0.0396 (9)	0.0608 (11)	−0.0005 (7)	0.0179 (8)	0.0021 (8)
C7	0.0520 (10)	0.0439 (10)	0.0702 (13)	0.0006 (8)	0.0301 (9)	−0.0017 (9)
C8	0.0487 (10)	0.0424 (10)	0.0641 (12)	−0.0002 (8)	0.0208 (9)	0.0022 (8)
C9	0.0596 (11)	0.0377 (9)	0.0693 (13)	−0.0040 (8)	0.0179 (9)	0.0027 (8)
C11	0.0500 (9)	0.0389 (9)	0.0586 (11)	−0.0006 (7)	0.0171 (8)	−0.0023 (8)
C12	0.0490 (9)	0.0453 (10)	0.0632 (11)	0.0006 (8)	0.0215 (8)	−0.0053 (8)
C13	0.0677 (12)	0.0437 (11)	0.0959 (16)	0.0051 (9)	0.0386 (11)	−0.0112 (10)
C14	0.0901 (15)	0.0365 (10)	0.120 (2)	0.0013 (10)	0.0539 (14)	−0.0020 (11)
C15	0.0716 (12)	0.0425 (11)	0.0944 (16)	−0.0034 (9)	0.0437 (12)	−0.0001 (10)
C16	0.0463 (9)	0.0421 (9)	0.0590 (11)	0.0023 (7)	0.0174 (8)	−0.0028 (8)
C17	0.0490 (10)	0.0458 (11)	0.0680 (12)	−0.0006 (8)	0.0253 (9)	−0.0024 (9)
C18	0.0459 (9)	0.0449 (10)	0.0550 (11)	0.0017 (7)	0.0156 (8)	−0.0055 (8)
C19	0.0565 (10)	0.0356 (9)	0.0757 (13)	0.0021 (8)	0.0175 (9)	−0.0074 (9)
C20	0.0687 (12)	0.0515 (12)	0.0933 (16)	0.0045 (10)	0.0290 (12)	−0.0180 (11)
N1	0.0482 (8)	0.0416 (8)	0.0667 (10)	−0.0036 (6)	0.0247 (7)	0.0024 (7)
N2	0.0621 (9)	0.0418 (9)	0.0857 (12)	−0.0043 (7)	0.0448 (9)	−0.0020 (8)
N3	0.0462 (8)	0.0434 (8)	0.0573 (9)	0.0035 (6)	0.0190 (7)	−0.0056 (7)
N4	0.0508 (8)	0.0408 (8)	0.0766 (11)	−0.0019 (6)	0.0308 (8)	−0.0107 (7)
O1	0.0744 (9)	0.0447 (7)	0.0956 (11)	−0.0005 (6)	0.0471 (8)	−0.0044 (7)
O2	0.0606 (7)	0.0382 (6)	0.0807 (9)	−0.0043 (5)	0.0344 (7)	0.0017 (6)
O3	0.0766 (9)	0.0517 (8)	0.0894 (10)	−0.0078 (6)	0.0513 (8)	−0.0038 (7)
O4	0.0623 (8)	0.0471 (7)	0.0762 (9)	−0.0043 (6)	0.0321 (7)	−0.0032 (6)
O5	0.0558 (7)	0.0389 (7)	0.0846 (9)	−0.0018 (5)	0.0320 (7)	−0.0125 (6)
O6	0.0766 (9)	0.0526 (9)	0.0970 (11)	−0.0015 (7)	0.0529 (8)	−0.0098 (7)

### Geometric parameters (Å, °)

**Table:**

C10—C9	1.496 (2)	C9—H9B	0.9700
C10—H10A	0.9600	C11—C12	1.382 (2)
C10—H10B	0.9600	C11—C16	1.386 (2)
C10—H10C	0.9600	C11—H11	0.9300
O1W—H1A	0.84 (3)	C12—O6	1.365 (2)
O1W—H1B	0.91 (3)	C12—C13	1.378 (3)
O2W—H2A	0.91 (3)	C13—C14	1.370 (3)
O2W—H2B	0.85 (3)	C13—H13	0.9300
O3W—H3A	0.89 (3)	C14—C15	1.380 (3)
O3W—H3B	0.90 (3)	C14—H14	0.9300
O4W—H4A	0.81 (3)	C15—C16	1.384 (2)
O4W—H4B	0.98 (6)	C15—H15	0.9300
C1—C2	1.382 (2)	C16—C17	1.456 (2)
C1—C6	1.386 (2)	C17—N3	1.270 (2)
C1—H1	0.9300	C17—H17	0.9300
C2—O3	1.359 (2)	C18—O4	1.212 (2)
C2—C3	1.379 (3)	C18—O5	1.3339 (19)
C3—C4	1.372 (3)	C18—N4	1.342 (2)
C3—H3	0.9300	C19—O5	1.443 (2)
C4—C5	1.378 (3)	C19—C20	1.494 (2)
C4—H4	0.9300	C19—H19A	0.9700
C5—C6	1.380 (2)	C19—H19B	0.9700
C5—H5	0.9300	C20—H20A	0.9600
C6—C7	1.458 (2)	C20—H20B	0.9600
C7—N1	1.271 (2)	C20—H20C	0.9600
C7—H7	0.9300	N1—N2	1.3759 (18)
C8—O1	1.206 (2)	N2—H2	0.8600
C8—O2	1.3303 (19)	N3—N4	1.3750 (18)
C8—N2	1.342 (2)	N4—H4N	0.8600
C9—O2	1.444 (2)	O3—H3C	0.8200
C9—H9A	0.9700	O6—H6	0.8200
			
C9—C10—H10A	109.5	O6—C12—C11	122.46 (16)
C9—C10—H10B	109.5	C13—C12—C11	120.25 (16)
H10A—C10—H10B	109.5	C14—C13—C12	119.85 (17)
C9—C10—H10C	109.5	C14—C13—H13	120.1
H10A—C10—H10C	109.5	C12—C13—H13	120.1
H10B—C10—H10C	109.5	C13—C14—C15	120.52 (18)
H1A—O1W—H1B	103 (3)	C13—C14—H14	119.7
H2A—O2W—H2B	103 (2)	C15—C14—H14	119.7
H3A—O3W—H3B	104 (3)	C14—C15—C16	119.99 (17)
H4A—O4W—H4B	109 (4)	C14—C15—H15	120.0
C2—C1—C6	120.13 (16)	C16—C15—H15	120.0
C2—C1—H1	119.9	C11—C16—C15	119.49 (15)
C6—C1—H1	119.9	C11—C16—C17	122.43 (15)
O3—C2—C3	117.57 (15)	C15—C16—C17	118.08 (15)
O3—C2—C1	122.59 (15)	N3—C17—C16	123.66 (15)
C3—C2—C1	119.83 (16)	N3—C17—H17	118.2
C4—C3—C2	120.04 (17)	C16—C17—H17	118.2
C4—C3—H3	120.0	O4—C18—O5	125.14 (16)
C2—C3—H3	120.0	O4—C18—N4	125.94 (15)
C3—C4—C5	120.39 (18)	O5—C18—N4	108.92 (14)
C3—C4—H4	119.8	O5—C19—C20	106.88 (14)
C5—C4—H4	119.8	O5—C19—H19A	110.3
C4—C5—C6	120.05 (17)	C20—C19—H19A	110.3
C4—C5—H5	120.0	O5—C19—H19B	110.3
C6—C5—H5	120.0	C20—C19—H19B	110.3
C5—C6—C1	119.53 (15)	H19A—C19—H19B	108.6
C5—C6—C7	118.03 (15)	C19—C20—H20A	109.5
C1—C6—C7	122.43 (15)	C19—C20—H20B	109.5
N1—C7—C6	123.50 (15)	H20A—C20—H20B	109.5
N1—C7—H7	118.3	C19—C20—H20C	109.5
C6—C7—H7	118.3	H20A—C20—H20C	109.5
O1—C8—O2	125.35 (16)	H20B—C20—H20C	109.5
O1—C8—N2	125.86 (16)	C7—N1—N2	114.59 (14)
O2—C8—N2	108.80 (14)	C8—N2—N1	120.80 (14)
O2—C9—C10	106.76 (14)	C8—N2—H2	119.6
O2—C9—H9A	110.4	N1—N2—H2	119.6
C10—C9—H9A	110.4	C17—N3—N4	114.73 (14)
O2—C9—H9B	110.4	C18—N4—N3	120.63 (14)
C10—C9—H9B	110.4	C18—N4—H4N	119.7
H9A—C9—H9B	108.6	N3—N4—H4N	119.7
C12—C11—C16	119.90 (16)	C8—O2—C9	117.01 (13)
C12—C11—H11	120.0	C2—O3—H3C	109.5
C16—C11—H11	120.0	C18—O5—C19	117.34 (13)
O6—C12—C13	117.29 (15)	C12—O6—H6	109.5
			
C6—C1—C2—O3	177.98 (18)	C12—C11—C16—C17	179.33 (18)
C6—C1—C2—C3	−1.1 (3)	C14—C15—C16—C11	0.1 (3)
O3—C2—C3—C4	−179.3 (2)	C14—C15—C16—C17	−179.6 (2)
C1—C2—C3—C4	−0.1 (3)	C11—C16—C17—N3	−7.5 (3)
C2—C3—C4—C5	0.7 (4)	C15—C16—C17—N3	172.2 (2)
C3—C4—C5—C6	−0.1 (4)	C6—C7—N1—N2	−178.90 (17)
C4—C5—C6—C1	−1.2 (3)	O1—C8—N2—N1	−0.5 (3)
C4—C5—C6—C7	178.4 (2)	O2—C8—N2—N1	179.68 (16)
C2—C1—C6—C5	1.8 (3)	C7—N1—N2—C8	−178.09 (19)
C2—C1—C6—C7	−177.82 (18)	C16—C17—N3—N4	179.20 (17)
C5—C6—C7—N1	−179.1 (2)	O4—C18—N4—N3	3.2 (3)
C1—C6—C7—N1	0.6 (3)	O5—C18—N4—N3	−177.24 (15)
C16—C11—C12—O6	−179.59 (17)	C17—N3—N4—C18	175.34 (18)
C16—C11—C12—C13	0.5 (3)	O1—C8—O2—C9	2.9 (3)
O6—C12—C13—C14	179.7 (2)	N2—C8—O2—C9	−177.33 (16)
C11—C12—C13—C14	−0.4 (3)	C10—C9—O2—C8	178.68 (17)
C12—C13—C14—C15	0.1 (4)	O4—C18—O5—C19	−2.9 (3)
C13—C14—C15—C16	0.0 (4)	N4—C18—O5—C19	177.54 (16)
C12—C11—C16—C15	−0.4 (3)	C20—C19—O5—C18	−171.15 (16)

### Hydrogen-bond geometry (Å, °)

**Table:**

*D*—H···*A*	*D*—H	H···*A*	*D*···*A*	*D*—H···*A*
N2—H2···O6^i^	0.86	2.29	3.0968 (18)	157
N4—H4N···O3	0.86	2.24	3.0849 (18)	167
O6—H6···O4W	0.82	1.90	2.675 (2)	157
C3—H3···O5	0.93	2.51	3.422 (2)	168
O1W—H1A···O2W^ii^	0.84 (3)	2.04 (3)	2.874 (3)	178 (3)
O1W—H1B···O2W^iii^	0.91 (3)	1.99 (3)	2.906 (2)	177 (3)
O2W—H2A···O4	0.91 (3)	2.02 (3)	2.899 (2)	163 (2)
O2W—H2B···O1^iv^	0.85 (3)	2.25 (3)	2.9268 (19)	136 (2)
O2W—H2B···N1^iv^	0.85 (3)	2.41 (3)	3.165 (2)	148 (2)
O3W—H3A···O4^iii^	0.89 (3)	2.27 (3)	2.9417 (19)	132 (2)
O3W—H3A···N3^iii^	0.89 (3)	2.38 (3)	3.200 (2)	153 (3)
O3W—H3B···O1	0.90 (3)	2.10 (3)	2.991 (2)	171 (3)
O4W—H4B···O3W^v^	0.98 (6)	1.84 (6)	2.819 (3)	173 (5)
O4W—H4A···O3W^iv^	0.81 (3)	2.15 (3)	2.955 (2)	169 (3)

Symmetry codes: (i) *x*−1, *y*, *z*+1; (ii) −*x*+1, −*y*+1, −*z*+1; (iii) −*x*+1, *y*−1/2, −*z*+3/2; (iv) −*x*+1, *y*+1/2, −*z*+3/2; (v) *x*+1, −*y*+1/2, *z*−1/2.
